# Activation Map‐Guided Ablation for Persistent Atrial Fibrillation Using Rhythmia Mapping System

**DOI:** 10.1002/joa3.70256

**Published:** 2025-12-17

**Authors:** Yosuke Murase, Yasuya Inden, Yasuhiro Ogawa, Hajime Imai, Naoaki Kano, Keita Mamiya, Katsuhiro Kawaguchi, Toyoaki Murohara

**Affiliations:** ^1^ Department of Cardiology Komaki City Hospital Komaki Aichi Japan; ^2^ Department of Cardiology Nagoya University Graduate School of Medicine Nagoya Aichi Japan

**Keywords:** activation map, catheter ablation, left atrial volume, organized atrial electrogram pattern, persistent atrial fibrillation

## Abstract

**Background:**

Creating activation map during atrial fibrillation (AF) has been challenging.

**Methods:**

Activation map‐guided ablation was performed in 29 persistent AF patients using the Rhythmia mapping system.

**Results:**

After activation map‐guided AF ablation, patients with left atrial volume (LAV) < 96.5 mL and ratio of organized atrial electrogram pattern (ROAE) ≥ 40% had significantly higher atrial arrhythmia‐free rates than other patients (84% vs. 20%, log‐rank test; *p* < 0.001).

**Conclusions:**

The patients with smaller LA and highly organized atrial electrogram pattern had significantly lower atrial arrhythmia recurrence rate after activation map‐guided AF ablation.

## Graphical abstract

Activation map‐guided ablation was performed in 29 persistent AF patients using the Rhythmia mapping system and all rotational and focal propagation sites received radiofrequency applications. The patients with smaller left atrium and highly organized atrial electrogram pattern had significantly lower atrial arrhythmia recurrence rate after activation map‐guided AF ablation.

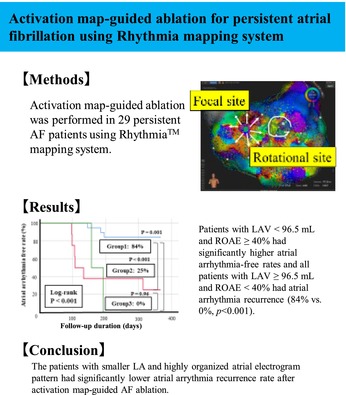

Mapping methods for visualizing the drivers of atrial fibrillation (AF) have recently been reported [1, 2]. To describe the drivers of AF, complicated atrial activation patterns, such as rotational and focal activation sequences, should be assessed in detail using a 3D‐mapping system. However, the efficacy of AF driver ablation has not been demonstrated in previous reports [3, 4]. It remains difficult to create activation map during AF because of complex activation sequence of atrial electrogram pattern. This study aimed to evaluate the outcome of activation map‐guided ablation for persistent AF using the Rhythmia mapping system.

Catheter ablation was performed in 29 patients with persistent AF using the Rhythmia mapping system at Komaki City Hospital between December 2021 and March 2023. To create the activation map, a 20‐pole circumferential catheter placed in the left atrial appendage (LAA) was used as the reference electrogram (EGM) after pulmonary vein isolation (PVI). The LAA was selected as the reference site because its EGM typically exhibited higher atrial signal amplitudes compared to those recorded from the coronary sinus, thereby enabling more reliable annotation during activation mapping. The cycle length (CL) curve during AF was measured using a reference EGM. The window of interest was set manually on the detected CL curve encompassing the dominant CL (Figure 1). We defined an organized atrial electrogram pattern as one in which the CL curve of the reference EGM was contained within the window of interest. When the reference EGM from the LAA was contained within this window, an atrial tachycardia‐like organized atrial electrogram pattern was obtained and reflected in the activation map. In contrast, when it was not, the wave was considered non‐organized atrial electrogram pattern and was not represented in the activation map. The ratio of organized atrial electrogram pattern (ROAE) was manually measured as the percentage of time (in seconds) during which the atrial electrogram pattern was contained within the window of interest over a 10‐s period of the CL curve derived from the LAA potential. After creating the LA activation map, local rotation and focal propagation sites were identified, and two or three adjacent radiofrequency applications were delivered (Figure 2). If rotational and focal sites were detected in the left atrial posterior wall, left atrial posterior wall isolation was performed.

**FIGURE 1:**
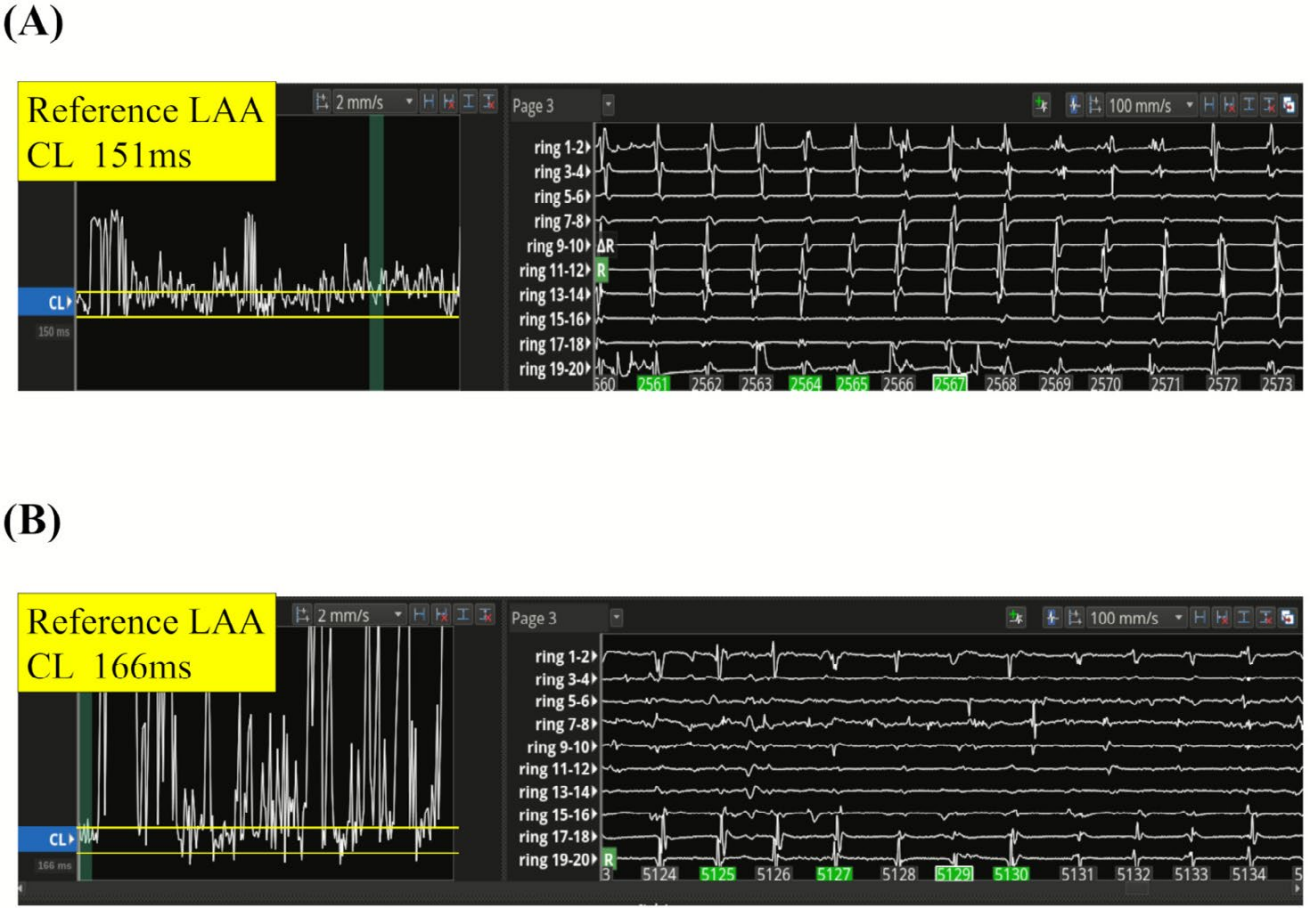
CL curve during AF measured using a reference bipolar EGM. The reference EGM was obtained using a 20‐pole circumferential catheter placed at the LAA. The window of interest was set manually on the detected CL curve encompassing the dominant CL (between the yellow lines). The CL curve of the organized atrial electrogram pattern almost covered the dominant CL (A). In contrast, the CL curve of the non‐organized atrial electrogram pattern did not match the coverage of the dominant CL (B). The ratio of organized atrial electrogram pattern (ROAE) was manually measured as the percentage of time (in seconds) during which the atrial electrogram pattern was contained within the window of interest over a 10‐s period of the CL curve derived from the LAA potential. AF, atrial fibrillation; CL, cycle length; EGM, electrogram; LAA, left atrial appendage; ROAE, ratio of organized atrial electrogram pattern.

**FIGURE 2:**
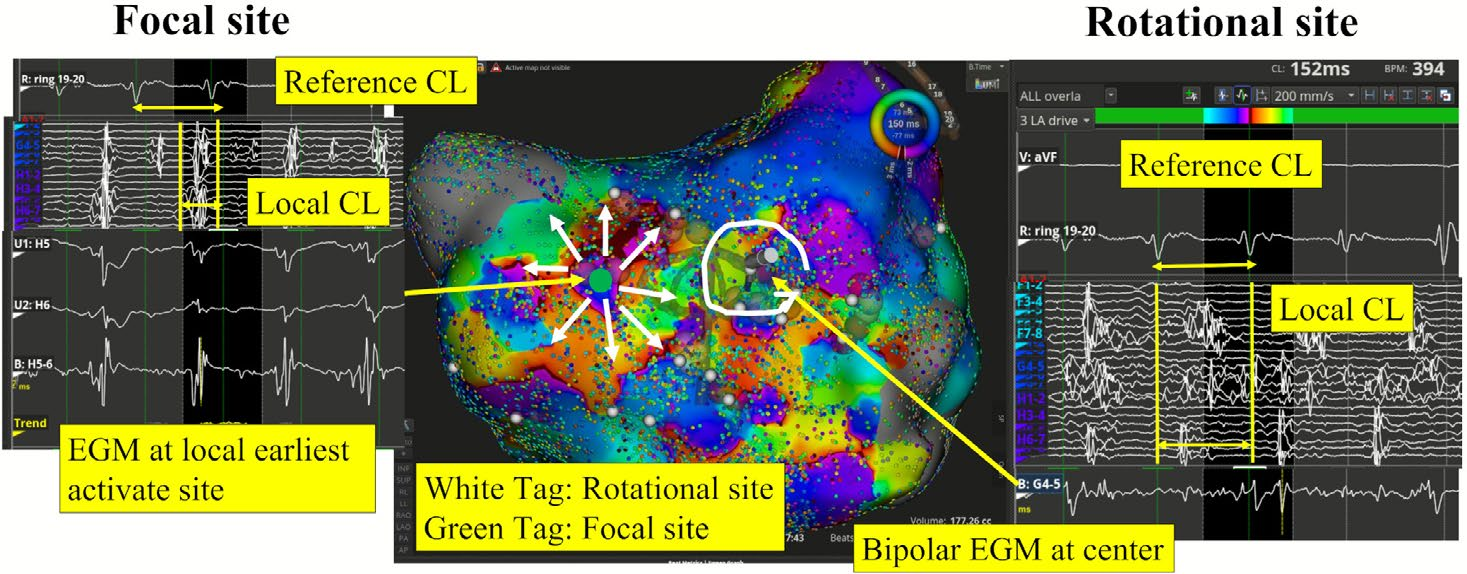
LA activation map during AF. The white tag shows the rotational site, and the green tag shows the focal site. Rotational site showing a rotational propagation pattern. Local EGM at the rotational site shows the dispersion electrical potential, including fractionated and non‐fractionated EGM. Local CL at the rotational site almost completely covered the reference CL. Bipolar EGM at the center of the rotational site showing a continuous low voltage EGM. In contrast, the focal site shows a centrifugal propagation pattern. Local EGM at the focal site shows the earliest activation site. Local CL at the focal site did not cover the reference CL. Unipolar EGM at the local earliest activation site shows the “RS” or “QS” pattern. AF, atrial fibrillation; CL, cycle length; EGM, electrogram; LA, left atrial.

At each follow‐up visit, patients underwent a 12‐lead ECG and 1‐week Holter monitoring was performed at 3, 6, and 12 months. All patients were followed up for 12 months after AF activation map‐guided ablation.

A comparison of the clinical characteristics of the patients with and without atrial arrhythmia recurrence is presented in Table 1. After activation map‐guided ablation, 11 (38%) patients experienced atrial arrhythmia recurrence during the follow‐up period. Patients with atrial arrhythmia recurrence had significantly larger left atrial volume (LAV) than the patients without atrial arrhythmia recurrence (LAV: 113.8 ± 21.7 mL vs. 80.1 ± 19.9 mL; *p* = 0.001). We set the cut‐off value of LAV as 96.5 mL (sensitivity, 73%; specificity, 89%; Figure 3).

**TABLE 1:** Comparison of clinical characteristics between the patients with and without atrial arrhythmia recurrence in the study population.

	All patients, *n* = 29	Atrial arrhythmia recurrence		*p*
		With, *n* = 11	Without, *n* = 18	
Parameters				
Age, year	73 ± 9	76 ± 6	71 ± 11	0.355
Male, *n* (%)	21 (72%)	8 (73%)	13 (72%)	0.976
Hypertension, *n* (%)	19 (66%)	8 (73%)	11 (61%)	0.523
Diabetes mellitus, *n* (%)	7 (24%)	4 (36%)	3 (17%)	0.229
History of heart failure, *n* (%)	6 (21%)	2 (18%)	4 (22%)	0.794
AF duration, months	37 ± 35	40 ± 33	35 ± 37	0.572
Longstanding persistent AF, *n* (%)	22 (76%)	8 (73%)	14 (78%)	0.758
History of AF ablation, *n* (%)	11 (38%)	6 (55%)	5 (28%)	0.149
BMI, kg/m^2^	23.5 ± 3.4	24.7 ± 3.0	22.8 ± 3.5	0.185
CHA_2_DS_2_‐VASc	3.1 ± 1.5	3.4 ± 1.3	2.9 ± 1.6	0.422
eGFR, mL/min/1.73m^2^	57.4 ± 17.7	48.6 ± 13.6	62.7 ± 18.0	0.028
NT‐proBNP, pg/mL	1031.6 ± 798.0	1047.1 ± 793.5	1023.4 ± 824.6	0.936
Echocardiography				
LVEF, %	60.7 ± 12.1	63.8 ± 10.9	58.8 ± 12.8	0.164
LAD, mm	43.8 ± 5.4	46.3 ± 3.6	42.2 ± 5.8	0.039
LA volume, mL	92.9 ± 26.2	113.8 ± 21.7	80.1 ± 19.9	0.001
e′	9.3 ± 2.6	9.0 ± 2.3	9.5 ± 2.7	0.719
Medication (post ablation)				
β‐blocker	13 (45%)	5 (46%)	8 (44%)	0.958
Class I	0 (0%)	0 (0%)	0 (0%)	
Class III	9 (31%)	4 (36%)	5 (28%)	0.628

Abbreviations: AF, atrial fibrillation; BMI, body mass index; eGFR, estimated glomerular filtration rate; LA, left atrial; LAD, left atrial diameter; LVEF, left ventricular ejection fraction; NT‐proBNP, N‐terminal pro‐brain natriuretic peptide.

**FIGURE 3:**
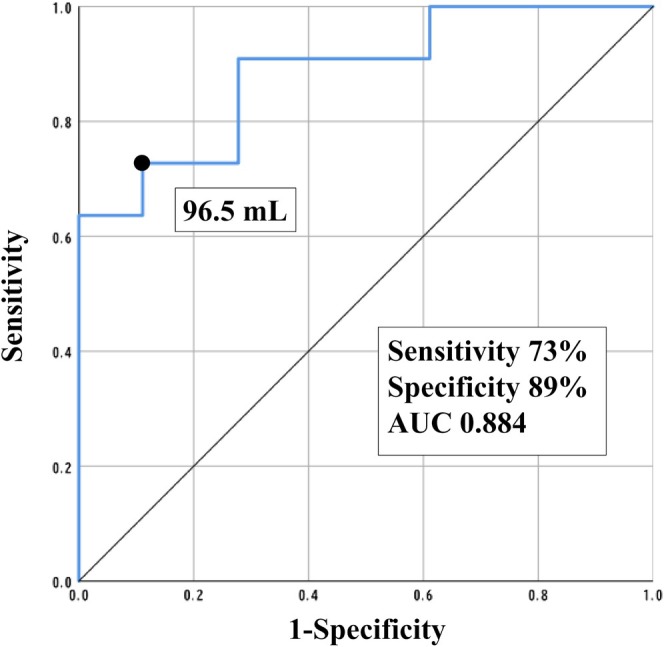
ROC curve of LAV for atrial arrhythmia recurrence after activation map‐guided ablation. AUC, area under the curve; LAV, left atrial volume; ROC, receiver operating characteristic.

A comparison of the details of activation map‐guided ablation in patients with and without atrial arrhythmia recurrence is presented in Table 2. The mean values of the rotational sites were 12 ± 6 points and the focal sites were 3 ± 2 points in the individual patients. The numbers of patients who underwent additional ablation did not differ significantly between the two groups. Figure 4 illustrates the distribution of rotational and focal sites within the left atrium. There were six patients (21%) with direct AF termination. None of the patients with direct AF termination experienced atrial arrhythmia recurrence during the follow‐up period.

**TABLE 2:** Comparison of the details of activation map‐guided ablation between the patients with and without atrial arrhythmia recurrence in the study population.

Parameters	All patients, *n* = 29	Atrial arrhythmia recurrence		*p*
		With, *n* = 11	Without, *n* = 18	
Mapping time, min	20 ± 8	20 ± 7	20 ± 8	0.369
Procedure time, min	211 ± 55	188 ± 52	226 ± 53	0.116
Mapping points, points	22 323 ± 7977	22 453 ± 6944	22 244 ± 8741	0.857
Reference CL, ms	161 ± 20	162 ± 19	161 ± 21	0.605
ROAE, %	74 ± 24	71 ± 24	76 ± 25	0.553
Rotational site, points	12 ± 6	12 ± 6	12 ± 7	0.77
Focal site, points	3 ± 2	4 ± 2	3 ± 2	0.177
RA driver ablation, *n* (%)	11 (38%)	4 (36%)	7 (39%)	0.892
CTI, *n* (%)	27 (93%)	10 (91%)	17 (94%)	0.715
Roof line, *n* (%)	13 (45%)	5 (46%)	8 (44%)	0.958
LAPWI, *n* (%)	8 (28%)	2 (18%)	6 (33%)	0.376
Mitral isthmus, *n* (%)	5 (17%)	1 (9%)	4 (22%)	0.364
AF termination during ablation, *n* (%)	6 (21%)	0 (0%)	6 (33%)	0.032

Abbreviations: AF, atrial fibrillation; CL, cycle length; CTI, cavo tricuspid isthmus; LAPWI, left atrial posterior wall isolation; RA, right atrial; ROAE, ratio of organized atrial electrogram pattern.

**FIGURE 4:**
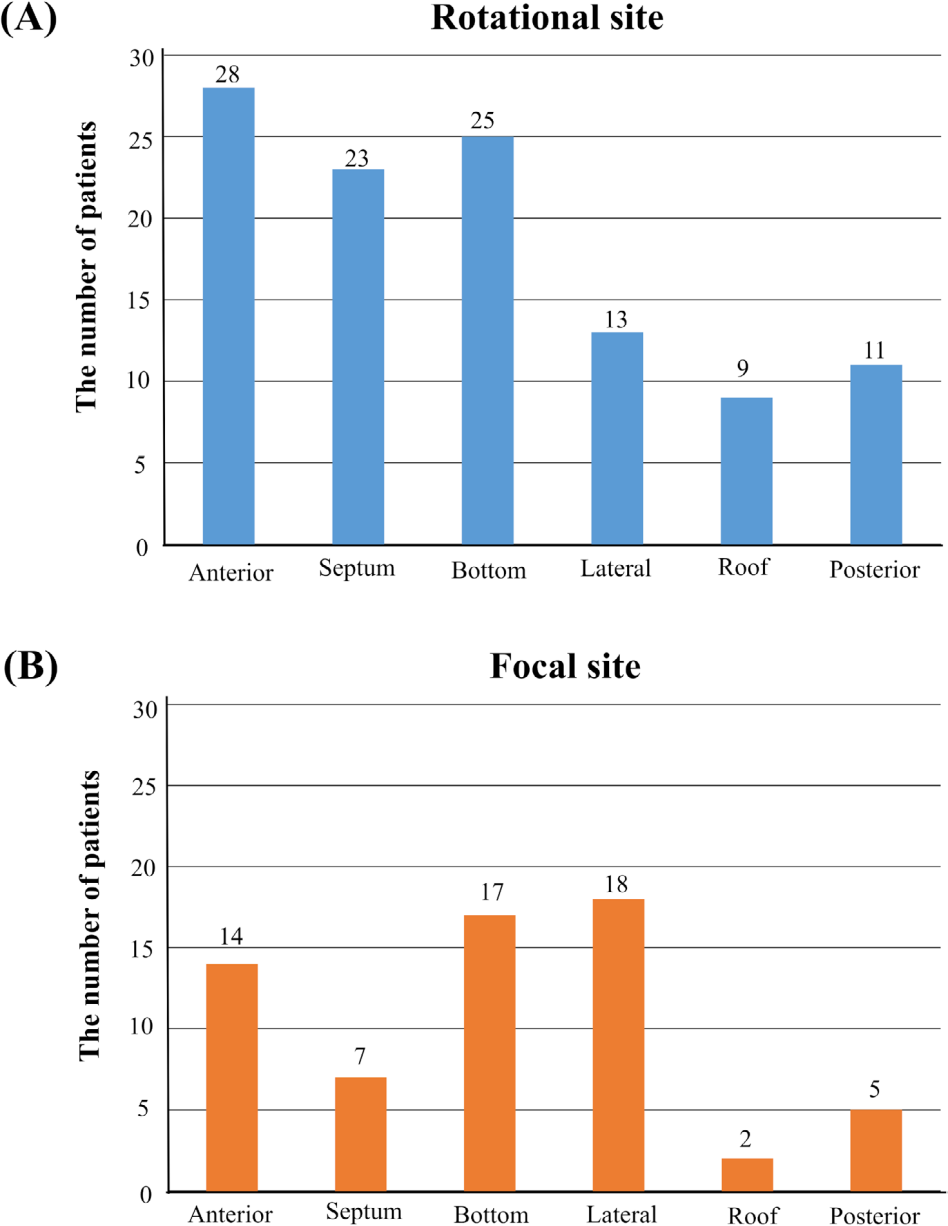
The distribution of rotational and focal sites within the left atrium. (A) Distribution of intracardiac locations of rotational sites. (B) Distribution of intracardiac locations of focal sites.

Figure 5 shows that patients with ROAE < 40% had higher atrial arrhythmia recurrence rates than patients with ROAE ≥ 40% (67% vs. 35%; Figure 5C) and did not show AF termination (0% vs. 23%; Figure 5F).

**FIGURE 5:**
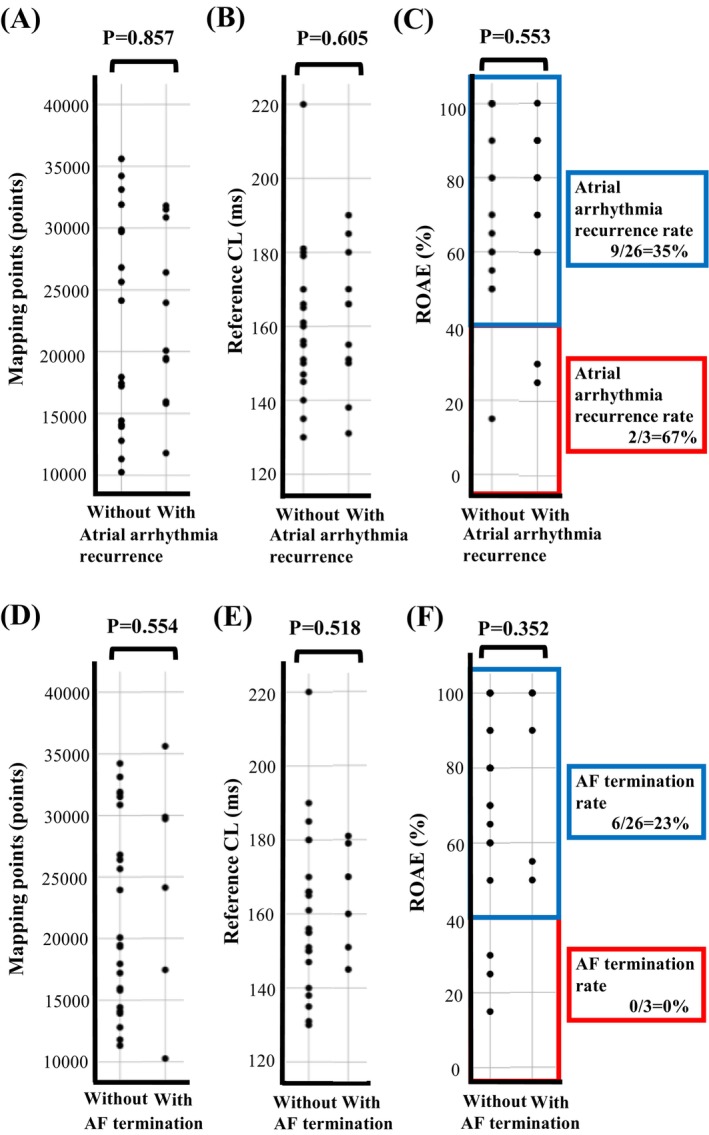
Scatter plots between atrial arrhythmia recurrence and the number of mapping points (A), reference CL (B), and ROAE (C). Additionally, scatter plots between AF termination during ablation and the number of mapping points (D), reference CL (E), and ROAE (F). AF, atrial fibrillation; CL, cycle length, ROAE, ratio of organized atrial electrogram pattern.

Kaplan–Meier analysis was performed to evaluate the risk factors for atrial arrhythmia recurrence after activation map‐guided ablation (Figure 6). Patients with LAV < 96.5 mL had significantly higher atrial arrhythmia‐free rates (84% vs. 20%, log‐rank test; *p* < 0.001; Figure 6A). Additionally, patients with LAV < 96.5 mL and ROAE ≥ 40% had significantly higher atrial arrhythmia‐free rates, and all patients with LAV ≥ 96.5 mL and ROAE < 40% had atrial arrhythmia recurrence (84% vs. 0%, *p* < 0.001; Figure 6B).

**FIGURE 6:**
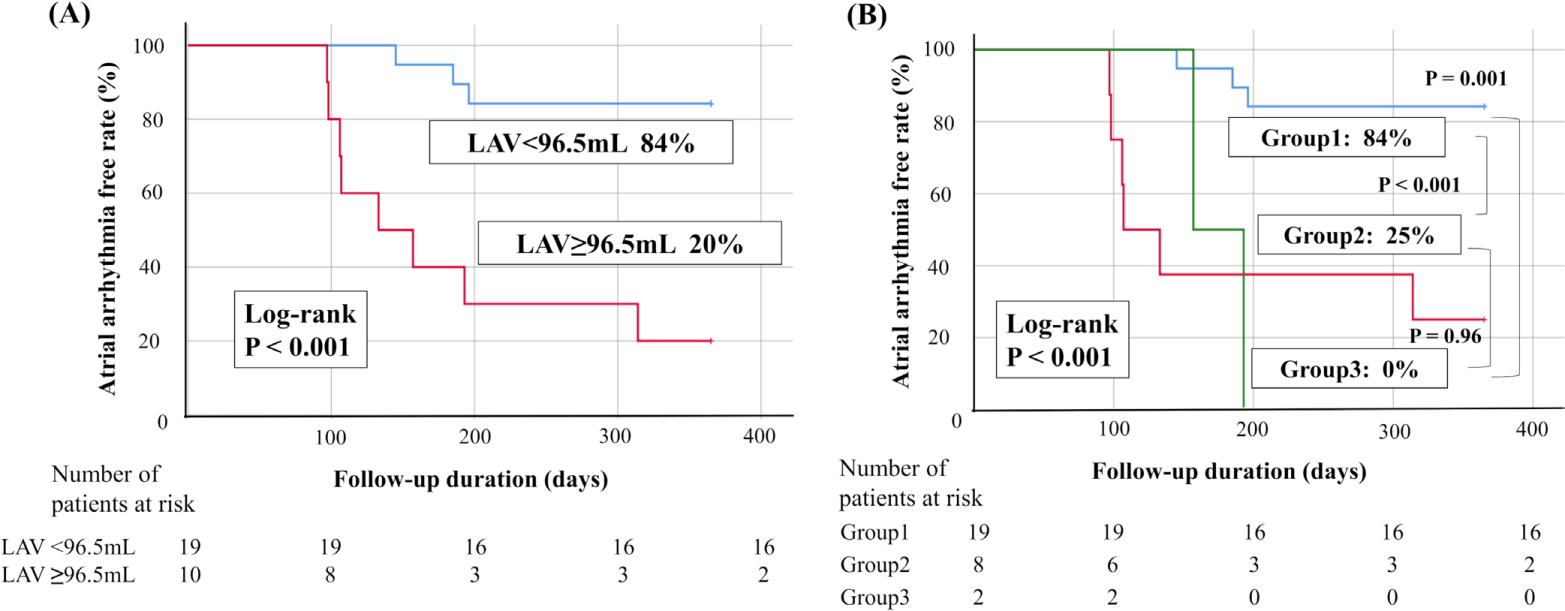
Kaplan–Meier curves of the incidence of atrial arrhythmia recurrence after driver‐guided ablation. (A) Patients with larger LAV (≥ 96.5 mL) had significantly lower atrial arrhythmia recurrence‐free rates. (B) All patients were divided into three groups (Group 1: LAV < 96.5 mL and ROAE ≥ 40%, Group 2: LAV ≥ 96.5 mL or ROAE < 40%, Group 3: LAV ≥ 96.5 mL and ROAE < 40%). Patients in Group 1 had significantly higher atrial arrhythmia‐free rates, and all patients in Group 3 had atrial arrhythmia recurrence. CL, cycle length; LAV, left atrial volume; ROAE, ratio of organized atrial electrogram pattern.

The main finding of this study was that the patients with smaller LA and highly organized atrial electrogram pattern had significantly lower atrial arrhythmia recurrence rate after activation map‐guided AF ablation. Activation map‐guided ablation for AF using the Rhythmia mapping system was previously reported by Lațcu et al. [5] In activation map‐guided ablation, the local activation propagation sites may represent potential AF driver sites. To our knowledge, our study is the first to demonstrate the patient characteristics associated with atrial arrhythmia recurrence after activation map‐guided ablation. Unlike CFAE ablation [6], which relies solely on electrogram signals, the activation map‐guided approach in this study incorporates activation mapping as an additional index, enabling more targeted ablation with a smaller lesion area. Similar to FIRM ablation [7], this method identifies rotational and focal propagation sites. A key advantage of activation map‐guided ablation using Rhythmia is its ultra‐high‐density mapping using a mini‐basket catheter with 64 mini‐electrodes (IntellaMap Orion; Boston Scientific), allowing for precise analysis of local potentials. As a result, the identified sites are more reliable than those in other AF driver analyses such as FIRM.

This study has several limitations. It was a single‐center study with a small sample size and lacked a control group undergoing PVI alone due to the absence of patients with comparable characteristics (e.g., AF duration), limiting assessment of efficacy. Additionally, it remains unclear whether this method is superior to previously reported beyond‐PVI approaches [8, 9, 10]. Larger multicenter studies are needed. Another concern is the reliability of the mapping technique. The visualization of the CL curve is unique to Rhythmia and not available in other systems like EnSite or CARTO. Although ROAE calculation is system‐based and less affected by inter‐operator variability, measurement timing and site identification are manual, potentially introducing subjectivity and operator‐dependent variability. These are inherent limitations of this technique.

## Funding

The authors have nothing to report.

## Conflicts of Interest

The authors declare no conflicts of interest.

## Data Availability

The data that support the findings of this study are available from the corresponding author upon reasonable request.
